# 

**Published:** 1822-06-01

**Authors:** 


### Contents

OF

No. 9, Vol. III, for JUNE, 1822.

ART. I.

BRITISH PATHOLOGY.

Dr. PARRY's Elements of Pathology and Therapeutics - - 1

II.

Dr. ASHBY SMITH's Edition of the Miscellaneous Works of Dr. Willan - - - - - - - - - - - - - - 41

III.

Mr. MARSHALL's Medical Topography of the Interior of Ceylon - - - - - - - - - - - - - - - - 53

IV.

Essays on Surgery and Midwifery. By Mr. JAMES BARLOW 64

V.

Mr. CLARKE on those Diseases of Females attended by Discharge. *(Art. 2d, Purulent Discharge.)* - - - - 77

VI.

Dr. EDWARD JENNER on the Influence of Artificial Eruptions in various Diseases - - - - - - - - - - - 89

VII.

Dr. CHISHOLM on the Climate and Diseases of Tropical Countries - - - - - - - - - - - - - - - - 100

VIII.

Dr. PRICHARD on Diseases of the Nervous System. Part I. Convulsive and Maniacal Affections. (First Analytical Article.) - - - - - - - - - - - - - - 122

IX.

*Lettera di Giacomo Clarke, &c.* A Letter from Dr. CLARK to Professor TOMMASINI, in Defence of the Edinburgh School of Medicine - - - - - - - - - - - - 151

X.

Mr. LLOYD on the Nature and Treatment of Scrophula. (First Analytical Article.) - - - - - - - - - - - - 156

### XI.

### Supplemental Review, or Quarterly Periscope of Practical Medicine and Surgery.

Introduction - - - - - - - - - - - - - - - 175
Art. 1. Mr. G. N. Hill on Tic Douloureux - - - - - - 176
2. Dr. Blicke on Cephalitis - - - - - - - - - - 178
3. Mr. Fielding on Fractured Patella - - - - - - 179
4. Mr. Cornish on Dislocated Femur - - - - - - 180
5. Traumatic Tetanus - - - - - - - - - - 181
6. M. Fouquier on Nervous Paralysis - - - - - - *ib.*
7. On Puncturing Anasarcous Limbs - - - - - - 182
8. Dr. Carson's Proposal in Phthisis - - - - - - 183
9. Mr. Coventry on Yellow Fever - - - - - - - 184
10. M. Fouquier on Obscure Pulmonary Disease - - 185
11. Oil of Croton-Tiglium - - - - - - - - - 186
12. Bath Waters - - - - - - - - - - - - - 188
13. Ascites cured by Pressure - - - - - - - - - 189
14. Ovarian Dropsy cured by Operation - - - - 190
15. Mr. Hume's Case of Poisoning - - - - - - - 192
16. Tympanites Intestinalis - - - - - - - - - - 193
17. Bronchocele, operated on - - - - - - - - - 194
18. Arteritis, Messrs. Bayle and Smerdon on - - - *ib.*
19. Inflammation of various Organs - - - - - - - 197
20. On the Identity of Variola and Varicella - - - 198
21. On Inflammatory Dropsy - - - - - - - - - 200
22. Mr. Stokes on Burns and Scalds - - - - - - 202
23. M. Husson on the Hepatic System - - - - - - *ib.*
24. Mr. Murray on Poisoning - - - - - - - - 203
25. Voltaic Battery - - - - - - - - - - - - 206
26. Edinburgh Surgical School - - - - - - - - 207
27. M. Breschet on Melanose - - - - - - - - 208
28. Mr. Barr on Tetanus - - - - - - - - - - - 209
29. Dr. Brown on Fractures of the Clavicle - - - - 211
30. Dr. Reed on Vaccine Protection - - - - - - - 213

### XII.

Bibliographical Record - - - - - - - - - - - - - 215

### XIII.

### MEDICAL INTELLIGENCE.

Art. 1. Royal College of Physicians of London - - - 219
2. Nux Vomica - - - - - - - - - - - - - - 220
3. Cubebs and Capivi - - - - - - - - - - - - 221
4. Sloughing Chancre - - - - - - - - - - - *ib.*
5. Crying of the Fœtus in Utero - - - - - - - *ib.*
6. Laurel Water - - - - - - - - - - - - - 222
7. The late Dr. Parry - - - - - - - - - - - *ib.*
8. A simple Argument not always inconclusive - - *ib.*
9. Vapour Baths - - - - - - - - - - - - - 223
10. Widow's Fund of Army Medical Officers - - - *ib.*

### XIV.

List of Subscribers - - - - - - - - - - - - - - 224

